# A Collection of Remarkable Cases of Surgery

**Published:** 1857-12

**Authors:** 


A Collection of Remarkable Cases in Surgery. By Paul F. Eve, Professor of Surgery in the Medical Department of the Nashville University. Published by J. B. Lippencott & Co. 1857. Pages 858. For sale by Keen & Lee, Chicago.

There is no book yet written in the profession so well calculated to excite and satisfy that universal quality of mankind, curiosity, as this production of the accomplished President of the American Medical Association. It is divided into ten chapters, taking the different parts of the body as a basis of division, beginning at the head. It is a perfect museum of the extraordinary.

It would seem that sometimes patients survive almost any mutilation or injury short of an entire removal of the head. Balls, nails, knives, ramrods, crowbars, breech pins of weapons may penetrate, pass through, or lodge in the brain, and yet the patient recover, with, as some of the French reporters naively observe, some "mental peculiarities." Dr. Eve has a section in chapter tenth called marvelous, but for fear of too nearly (for the publishers' sake) satisfying the curiosity of the reader, we shall leave him to infer the description of cases contained in it. The hints we have given him of those quite common cases of injuries of the brain, in first chapter, will doubtless enable him to do so without any great exertion of the imagination. It would be superfluous to recommend everybody to read it.

